# p63 Attenuates Epithelial to Mesenchymal Potential in an Experimental Prostate Cell Model

**DOI:** 10.1371/journal.pone.0062547

**Published:** 2013-05-01

**Authors:** Jan Roger Olsen, Anne Margrete Oyan, Kari Rostad, Margrete R. Hellem, Jie Liu, Lisha Li, David R. Micklem, Hallvard Haugen, James B. Lorens, Varda Rotter, Xi-Song Ke, Biaoyang Lin, Karl-Henning Kalland

**Affiliations:** 1 The Gade Institute, University of Bergen, Bergen, Norway; 2 Department of Microbiology, Haukeland University Hospital, Bergen, Norway; 3 Zhejing-California International NanoSystems Institute, Zhejiang University, Hangzhou, P.R. China; 4 BerGenBio AS, Bergen, Norway; 5 Department of Biomedicine, University of Bergen, Bergen, Norway; 6 Department of Molecular Cell Biology, Weizmann Institute of Science, Rehovot, Israel; 7 Swedish Medical Center, Seattle, Washington, United States of America; 8 Department of Urology, University of Washington, Seattle, Washington, United States of America; Ghent University, Belgium

## Abstract

The transcription factor p63 is central for epithelial homeostasis and development. In our model of epithelial to mesenchymal transition (EMT) in human prostate cells, p63 was one of the most down-regulated transcription factors during EMT. We therefore investigated the role of p63 in EMT. Over-expression of the predominant epithelial isoform ΔNp63α in mesenchymal type cells of the model led to gain of several epithelial characteristics without resulting in a complete mesenchymal to epithelial transition (MET). This was corroborated by a reciprocal effect when p63 was knocked down in epithelial EP156T cells. Global gene expression analyses showed that ΔNp63α induced gene modules involved in both cell-to-cell and cell-to-extracellular-matrix junctions in mesenchymal type cells. Genome-wide analysis of p63 binding sites using ChIP-seq analyses confirmed binding of p63 to regulatory areas of genes associated with cell adhesion in prostate epithelial cells. DH1 and ZEB1 are two elemental factors in the control of EMT. Over-expression and knock-down of these factors, respectively, were not sufficient alone or in combination with ΔNp63α to reverse completely the mesenchymal phenotype. The partial reversion of epithelial to mesenchymal transition might reflect the ability of ΔNp63α, as a key co-ordinator of several epithelial gene expression modules, to reduce epithelial to mesenchymal plasticity (EMP). The utility of ΔNp63α expression and the potential of reduced EMP in order to counteract metastasis warrant further investigation.

## Introduction

A family of transcription factors is constituted by p53, p63 and p73. Unlike p53, which is expressed in response to environmental stress, p63 (*TP63*) is constitutively expressed at high levels in a variety of epithelial tissues including prostate. There are two different promoters leading to two N-terminal variants ΔN- and TAp63 [1] and five reported splice variants of the C-terminal (α, β, γ, δ and ε) [2]. Originally, only the TA isoforms were considered to be transcriptional activators, but the consensus is now that ΔNp63 isoforms that carry a truncated aminoterminal domain have transcriptional activity not only restricted to the TAp63 controlled genes [1].

Mice with p63 knock-out fail to develop a prostate, highlighting p63’s important role in development. In adult tissues p63 is normally expressed in basal cells of the prostate and other stratified epithelia, but the expression is lost in more differentiated luminal cells. In prostate cancer cells p63 is typically undetectable and this is the basis for routine immunohistochemistry diagnostics of this cancer type. In benign prostate hyperplasia p63 positive cells are still numerous, leading to high cancer sensitivity for negative p63 staining of suspected prostate cancers [3]. Despite these insights, the role of p63 in differentiation of prostate cells is poorly understood, although mouse studies have shown that secretory cells in the prostate gland are derived from previously p63 positive cells [4]. There are also many unresolved questions regarding the cell of origin of prostate cancer and the role of p63, with some reports suggesting p63-positive basal cells [5] and others Nkx3-1-positive luminal cells [6] as the cell of origin. Additionally, the ΔNp63α isoform has been implicated as an oncogene in several types of squamous cell carcinomas, while other lines of evidence suggest that p63 functions in a tumor suppressive manner [1].

The epithelial to mesenchymal transition (EMT) is a preserved cellular program used extensively during embryogenesis. It has also been shown to be utilized in the adult organism notably in the processes of fibrosis and carcinogenesis. In carcinogenesis EMT is implicated in gain of invasiveness and escape from the primary site, an early event in metastasis [7].

We have previously published that the immortalized primary epithelial cells, EP156T, underwent EMT during long-term culture and selection for cells with loss of cell contact inhibition [8]. The EPT1 and EPT2 cells that were derived from EP156T cells following EMT have maintained their mesenchymal like morphology for more than 100 subsequent sub-confluent passages during the last 4 years [9].

EMT can be induced by forced expression of certain classical mesenchymal transcription factors such as ZEBs, SNAIs and TWISTs or knock-down of E-Cadherin [10]. Much less investigaton has been done regarding putative epithelial transcription factors, but recently transcription factors responsible for maintaining and inducing the epithelial state, such as GRHL2, ELF3 and ELF5 have been acknowledged [11]. Recently, p63 has been shown to play a role in EMT of breast cells [12]. In the present work we have investigated the potential of p63 as an epithelial transcription factor in prostate cells. We found that p63 is lost during EMT of prostate EP156T cells and that re-expression of its predominant isoform ΔNp63α in the mesenchymal type cells re-introduces several epithelial characteristics, suggesting an important role during EMT.

## Results

### ΔNp63α is the predominant isoform in EP156T cells and is shut down during EMT

**Figure 1:**
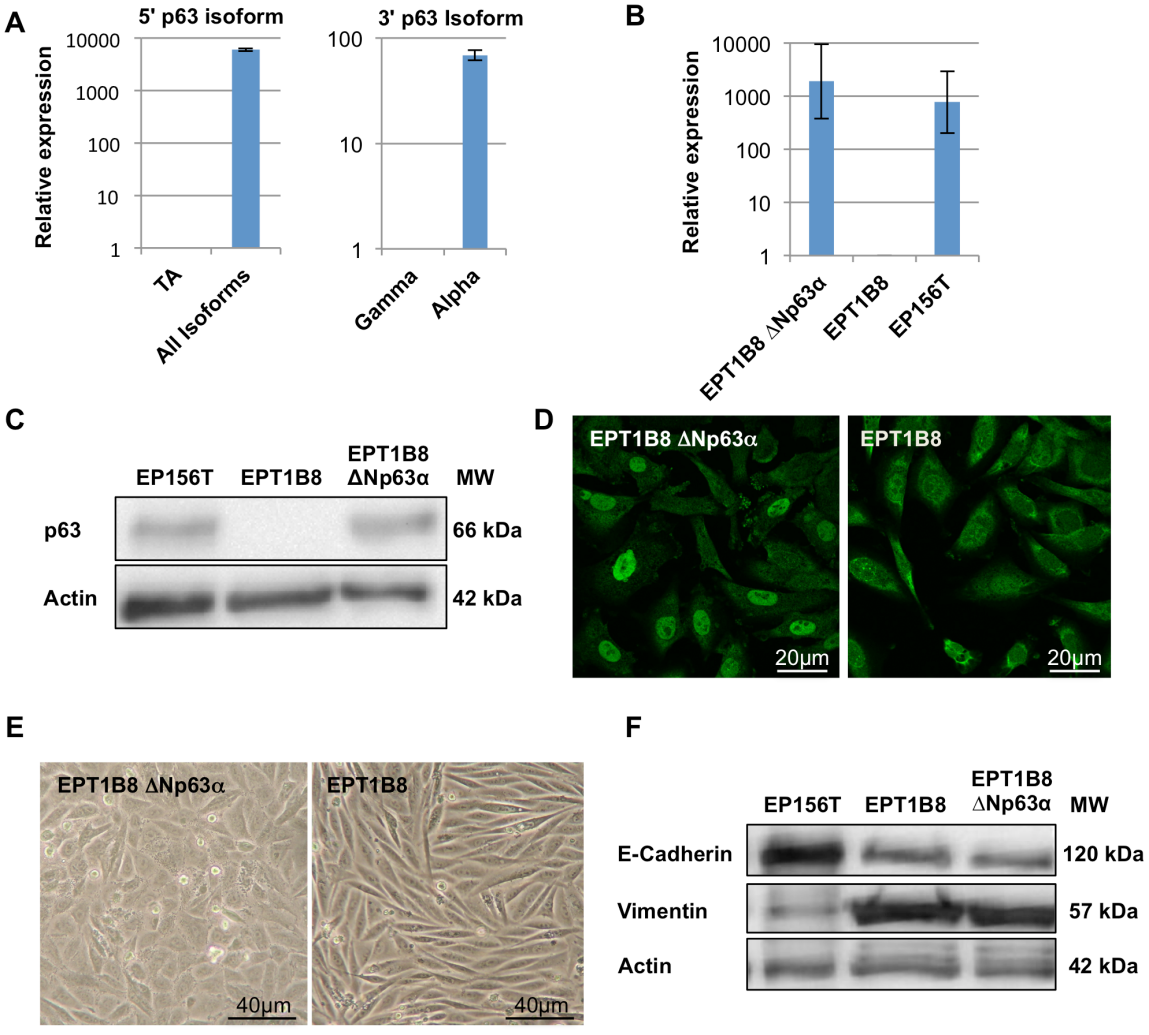
ΔNp63α over-expression in EPT1B8 cells and induction of epithelial features. (A) RT qPCR of EP156T cells, TA isoforms are low abundance compared to all isoforms, and α-isoforms are much more enriched than γ-isoforms, suggesting that ΔNp63α is the most expressed isoform. (B) RT qPCR and (C) Western Blot stained with p63 and actin show that ΔNp63α is over-expressed in EPT1B8 cells to similar levels as EP156T cells (D) Immunofluorescence in EPT1B8 ΔNp63α and EPT1B8 with p63 show nuclear staining in EPT1B8 ΔNp63α. Error bars show ±s.d. (E) Phase contrast microscopy of EPT1B8 ΔNp63α and EPT1B8. (F) Western Blot of EP156T, EPT1B8 ΔNp63α and EPT1B8 cells using antibodies against vimentin and E-Cadherin.

We have previously published that EMT is one part of the stepwise transformation model based on human primary immortalized, epithelial prostate cells [8], [9]. Genome-wide gene expression analyses revealed that p63 was one of the most down-regulated transcription factors when epithelial EP156T cells underwent EMT to become EPT1 cells. This led us to question if p63 has a direct role in the process of EMT and its reversibility in prostate cells. To further investigate the role of p63, we performed isoform-specific reverse transcriptase quantitative PCR (RT-qPCR) of EP156T cells. This demonstrated that ΔNp63α was the predominant isoform (Figure 1A), in line with earlier reports showing that ΔNp63α is the most expressed isoform in basal prostate cells [13].

Based on these data we decided to express ΔNp63α in EPT1, EPT2 and EPT1B8 cells, the latter is a clone of the published EPT1 cells. The ΔNp63α gene was recombined into a retroviral vector (Figure S1B) and transduced into EPT mesenchymal type cells, lacking detectable endogenous p63. Re-expression of p63 to levels comparable to expression in EP156T cells was verified using RT qPCR and Western blot analyses, while indirect immunofluorescence analysis of p63 showed nuclear staining (shown for EPT1B8 in Figure 1B, 1C, 1D). The possible mesenchymal-to-epithelial transition (MET) was assayed using whole-genome gene expression microarray data and evaluation of cell morphology.

Re-expression of ΔNp63α did not lead to a complete reversion of morphological features comparable to those seen in EP156T in monolayer cultures, but the mesenchymal EPT cells re-expressing ΔNp63α were markedly changed from the mock transduced cells, particularly at confluent growth, as the ΔNp63α transduced cells failed to organize like long spindle-shaped cells, but organized themselves towards an epithelial cobblestone pattern (Figure 1E). Western blot analysis of E-Cadherin and vimentin expression is often used as a marker of either an epithelial or mesenchymal state. EPT1B8 cells re-expressing ΔNp63α did not show any altered E-Cadherin or vimentin protein expression compared to EPT1B8 cells (Figure 1F).

### Re-expression of ΔNp63α in mesenchymal cells induces expression of genes involved in cell-to-cell junctions and extracellular matrix (ECM) contacts

**Figure 2:**
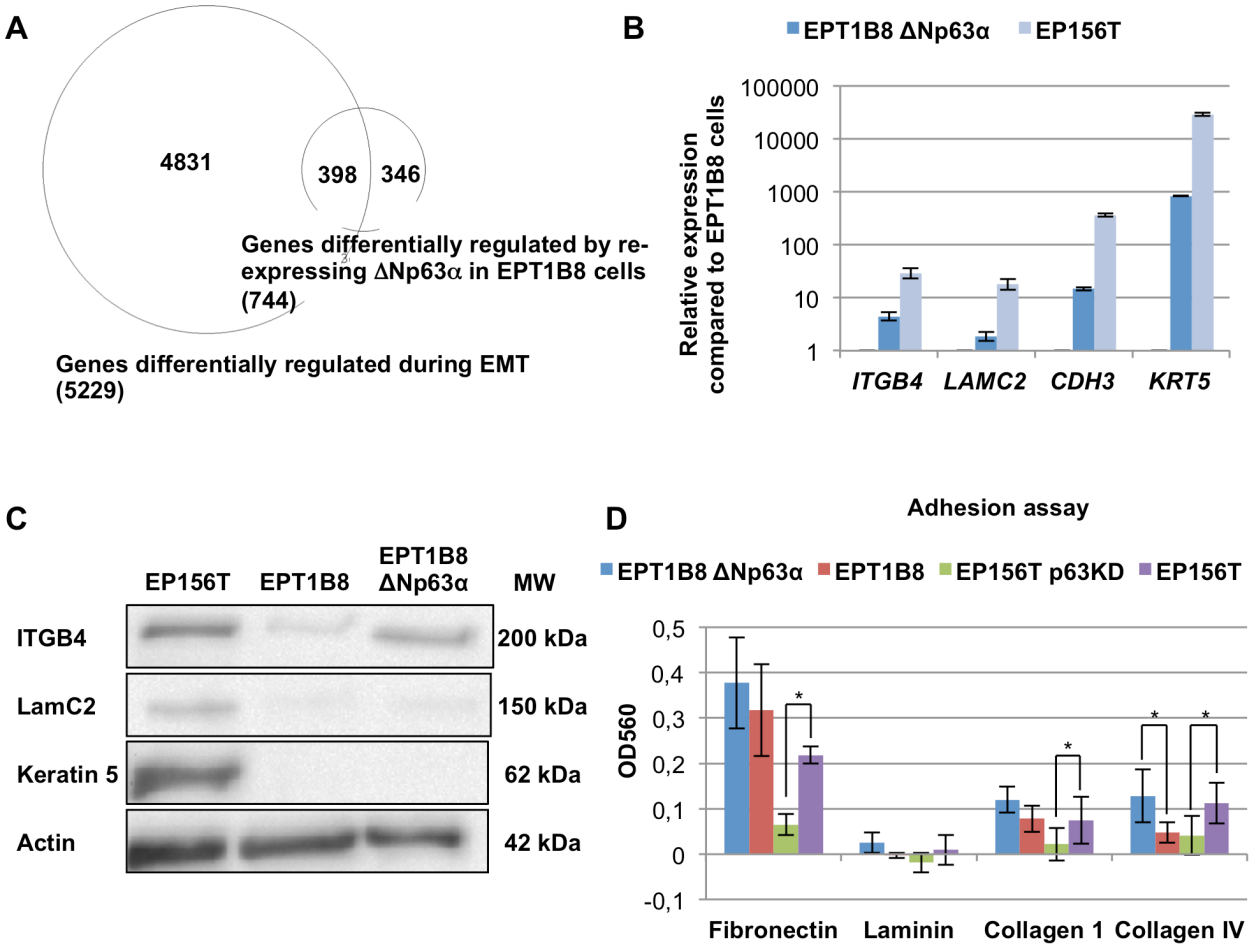
ΔNp63α re-expression induces cell junction modules in EPT1B8 cells. (A) Venn diagram of genes differentially expressed with an FDR <5% and fold change >2 in EMT (EPT1B8 compared to EP156T) and after ΔNp63α re-expression in EPT1B8. (B) RT qPCR and (C) Western blot of genes involved in cell adhesion ITGB4, LAMC2, CDH3 and KRT5 in EPT1B8 ΔNp63α and EPT1B8 compared to EP156T cells. Error bars show ±s.d. (D) EPT1B8, EPT1B8 ΔNp63α, EP156T and EP156T p63 knock-down were grown on different extracellular matrix substrates. Error bars show ±s.d. of at least three replicates. Student’s t-test was used for statistical analysis (*, p<0.05).

We previously published that multiple gene module switches were observed when EP156T cells underwent EMT and became EPT1 cells during long-term confluent culture, including cell junction, cell polarity, cytoskeleton, receptor tyrosine kinase, transcription factor and microRNA modules [8] (Figure S1C). This model therefore offers an excellent opportunity to examine the requirements for reversal of EMT, *i.e.* to examine if single master regulators have the power to co-ordinate the entire EMT/MET program or whether reversal of selected modules can create intermediate stages between the epithelial and the mesenchymal states. To investigate if ΔNp63α can coordinate gene modules relevant to EMT, we performed genome-wide microarray gene expression analysis of EPT1B8 cells transduced with ΔNp63α compared with mock-transduced cells. Significance Analysis of Microarray (SAM) showed that 163 genes were down- and 581 genes were up-regulated at least 2 fold at the highest False Discovery Rate (FDR <5%). Still, the number of genes differentially expressed in EPT1B8 cells after ΔNp63α re-expression were 744 compared to 5229 genes differentially expressed after EMT from EP156T cells to EPT1B8 cells, *i.e.* 14.2% of the genes are re-expressed in mesenchymal EPT1B8 ΔNp63α re-expressing cells (Figure 2A). 398 genes were shared between the groups, showing highly significant (p<0.0001, Chi-square test) enrichment of genes regulated by ΔNp63α re-expression among differentially expressed genes during EMT. We then wanted to see if ΔNp63α regulates specific gene modules. For this purpose we evaluated gene ontology terms and the differential expression of their constituent genes based on microarray findings. 72 of the 737 genes in the biological adhesion group (GO:0022610) that were represented on the array were differentially regulated compared to a total of 744 differentially expressed genes out of 25988 genes in the dataset (p<0.0001, Fisher’s exact test). The same relation was found for ”cell junction”, ”gap junction”, ”desmosome”, ”adherens junction” and ”cell-cell junction” (Table 1). Using RT qPCR we verified the differential regulation of several cell adhesion genes based on the microarray data. Although multiple cell adhesion genes were up-regulated, the expression levels did not reach that of EP156T cells (Figure 2B), as also confirmed at the protein level (Figure 2C).

**Table 1:** Enrichment of cell junction terms in EPT1B8 ΔNp63α.

CELL ADHESION GROUP	GENE ONTOLOGY ID	DIFFERENTIALLY EXPRESSED GENES	GENES IN GROUP IN DATASET	P-VALUE
Biological adhesion	GO:0022610	72	737	<0.0001
Cell junction	GO:0030054	56	635	<0.0001
Gap junction	GO:0005921	3	20	0.0185
Desmosome	GO:0030057	5	22	0.0003
Adherens junctions	GO:0005912	8	74	0.0012
Cell-cell junction	GO:0005911	18	160	<0.0001
Focal adhesion	GO:0005925	12	122	0.0002
Tight Junction	GO:0005923	9	94	0.0015
Cell-matrix adhesion	GO:0007160	7	95	0.0195
Hemisdesmosome	GO:0030056	2	8	0.0205

Relative enrichment of GO-terms related cell adhesion in EPT1B8 ΔNp63α compared to EPT1B8, using specific search for terms for different cell adhesion complexes. P-values are nominal and calculated by Fischer’s exact test.

To functionally examine the possibility that ΔNp63α expression affects cell adhesion we grew the cells on an array of ECM components and found stronger binding to ECM components for ΔNp63α re-expressing cells although only differential binding to collagen IV was statistically significant (Figure 2D).

### ΔNp63α re-expression reverses several phenotypic traits associated with EMT

**Figure 3:**
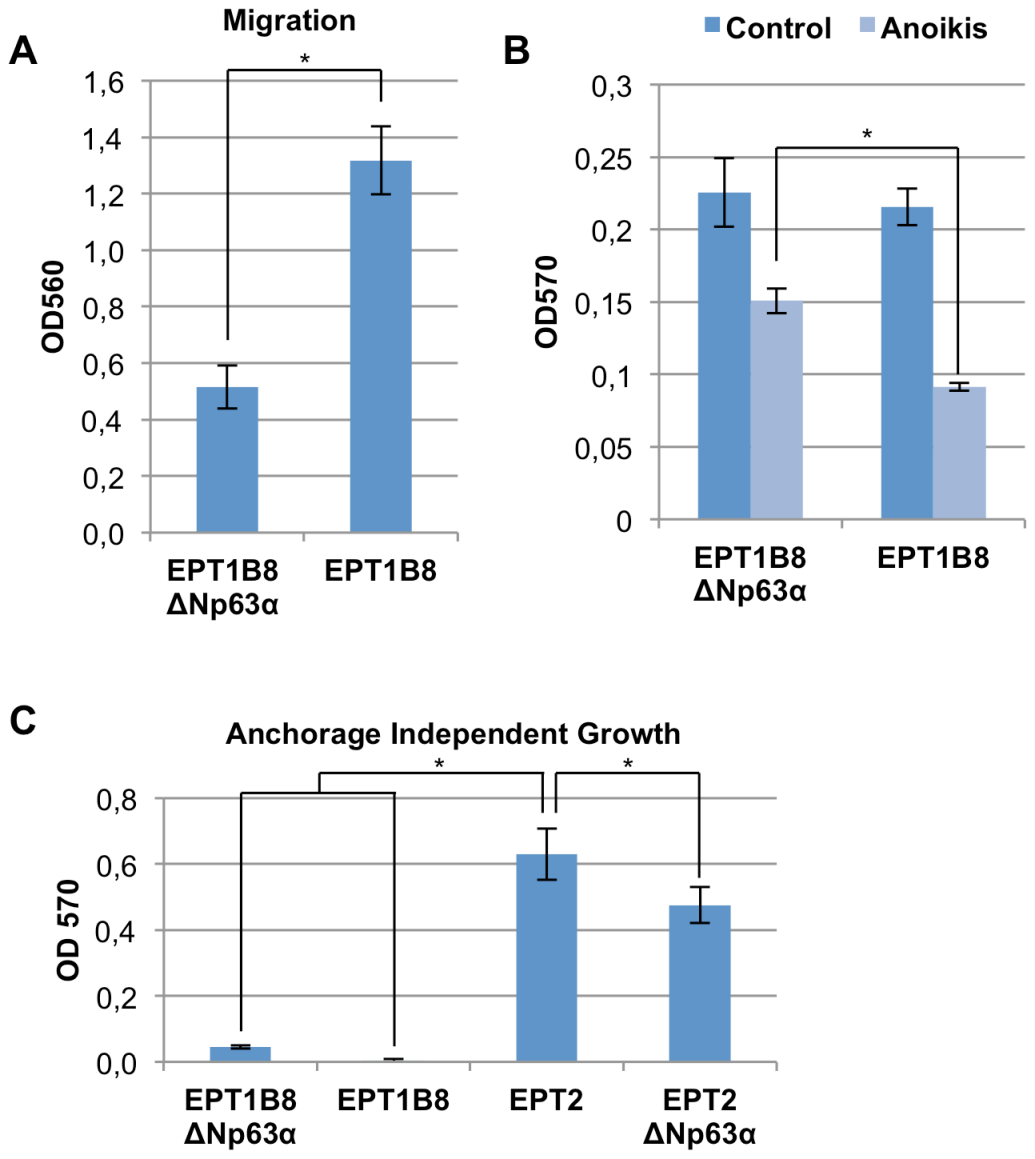
Functional changes after ΔNp63α over-expression in EPT1B8 cells. (A) Boyden chamber migration assay of EPT1B8 ΔNp63α and EPT1B8 cells. (B) EPT1B8 ΔNp63α and EPT1B8 cells grown on hydrogel covered wells (anoikis) and regular wells, cells alive stained after 24 hours. (C) Anchorage independent growth in soft-agar for EPT1B8 ΔNp63α, EPT1B8, EPT2 and EPT2 ΔNp63α. Error bars show ±s.d of at least three replicates. Student’s t-test was used for statistical analysis (*, p<0.001).

We further characterized the EPT1B8ΔNp63α cells concerning traits associated with epithelial cells. Following EMT, cells have increased migratory ability. We therefore assessed the ability of EPT1B8 cells to migrate in a Boyden chamber assay after ΔNp63α re-expression. Indeed, the EPT1B8ΔNp63α cells had a markedly and statistically significant (p<0.0001, Student’s t-test) decreased ability to migrate (Figure 3A). We also investigated if the ability to invade was altered, by a similar assay where the cells had to pass a pore with a membrane of extracellular matrix (ECM). The EPT1B8 cells re-expressing ΔNp63α did, however, not show any significant difference of invasive behavior (Figure S2A).

p63 has previously been implicated in resistance to anoikis in mammary epithelial cells [14], we therefore sought to find out if ΔNp63α plays a similar role in prostate cells. EPT1B8ΔNp63α and EPT1B8mock cells were seeded in wells covered with a hydrogel coating, which inhibits adhesion to the substrate, after 48 hours we observed a statistically significant higher amount of living cells among ΔNp63α expressing cells based on the MTT proliferation assay. Cells growing on a normal substrate served as control. Here ΔNp63α expressing cells yielded the same values as the control cells, thus ruling out that difference in proliferation could cause the observed difference in the anoikis assay (Figure 3B). Increased resistance to apoptosis is another trait associated with EMT. When cells were treated with the apoptosis-inducing agent staurosporine we could not detect any significant difference in the amount of activated caspase 3/7, as a marker of apoptosis (Figure S2B).

Cells with a mesenchymal phenotype are characterized by actin stress fibers as opposed to epithelial cells that display a cortical actin organization. ΔNp63α re-expression in EPT1B8 cells (and EPT1 and EPT2) did not reverse the stress fibers observed in mesenchymal cells (Figure S2C).

As p63 has been implicated in carcinogenesis we also wanted to investigate the effect of p63 on growth in soft agar, which has been considered an *in vitro* transformation assay [15]. ΔNp63α has been found to be over-expressed in several carcinomas [1]. EPT1 cells have already gained several traits associated with cancer cells as compared to the epithelial EP156T cells [8], and we further wanted to explore if expression of ΔNp63α could endow EPT1B8 cells with the ability to grow anchorage independently, or abolish the ability of EPT2 cells to do so [9]. Compared to EPT2 cells that have this ability, ΔNp63α did not provide EPT1B8 cells with this ability. EPT2ΔNp63α cells did exhibit a small but significantly decreased ability to grow in soft-agar (Figure 3C).

### Knock-down of p63 in EP156T cells

To complement over-expression studies of ΔNp63α in EPT1B8 cells we wanted to see if abrogation of p63 expression could induce EMT or reverse selected epithelial features in EP156T cells that contain high levels of endogenous p63. We transduced EP156T cells with shRNA particles targeting three different regions of the DNA-binding domain of p63, thereby targeting all isoforms. Knock-down was confirmed by RT qPCR and Western blot assays (Figures S3A, S3B).

Following p63 knock-down we could not observe any alterations in cell morphology in monolayer cultures (data not shown), but when analyzed by genome-wide gene expression analysis we found more genes differentially expressed than we found after ΔNp63α re-expression in EPT1B8 cells. 1408 genes were down-regulated and 1220 genes up-regulated at least 2 fold at a FDR <5%. Several of the cell adhesion gene modules were significantly differentially expressed, for example “Cell Junction”, “Focal adhesion” and “Tight Junction” (Table S1). We also validated differentially expressed adhesion genes by RT qPCR (Figure S3C). The ECM adhesion assay showed that EP156T p63 knock-down cells had significantly decreased binding to fibronectin, laminin and collagen IV while collagen I also exhibited decreased binding (p = 0.05, Student’s t-test), demonstrating complimentary results compared to the ΔNp63α over-expression in EPT1B8 cells (Figure 2D).

We then performed phenotypic assays on the EP156T cells with p63 knock-down as for the p63 re-expression in EPT1B8 cells. We did not find any significant changes in the ability to migrate (Figure S3D), but the EP156T p63 knock-down cells had a small, but statistically significant decreased ability to invade compared to EP156T cells (Figure S3E). The EP156T p63 knock-down cells also displayed a significantly decreased sensibility to the apoptosis-inducing agent staurosporine (Figure S3F). We also observed a significant decrease in MTT-based proliferation activity of EP156T p63 knock-down cells compared to control when grown on a hydrogel coating, suggesting increased anoikis-sensibility, although this could also be the result of higher proliferation as the MTT value was higher for vector-transduced EP156T cells (Figure S3G).

### p63 is able to co-ordinate induction of cell junction modules in mesenchymal prostate cells

In EPT1B8 cells the re-expression of ΔNp63α led to the induction of multiple cell junction components. The co-ordinated gene expression induction is exemplified by hemidesmosome components, including the extracellular LAMC2, its integrins ITGA6 and ITGB4 and its associated cytoplasmic cytokeratins, such as KRT5 (Figure 2B). It is interesting to know if these genes are direct target genes of p63 in order to understand how p63 can execute a co-ordinated gene expression program in both the epithelial and mesenchymal contexts. Carroll *et al*. used ChIP-PCR to demonstrate direct binding of p63 in the promoters of ITGA6, ITGB4 and LAMC2 in breast epithelial cells [14].

In order to identify p63 binding genes, we performed genome-wide profiling of p63 binding in our prostate cell model, EP156T cells using the ChIP-seq technology [16], [17]. ChIP-grade anti-p63 antibody was used for chromatin immunoprecipitation followed by deep sequencing. In the end, we obtained 20,511,592 raw reads from the ChIP with anti-p63 antibody and 19,455,454 raw reads from ChIP with the mouse IgG antibody. The sequence tags were mapped to the human genome (hg19) with the bowtie algorithm [18] using default parameters. After mapping, we obtained 14,868,166 and 13,494,179 uniquely mapped reads. We used MACS (model based analysis for ChIP-seq) [19] to find p63 binding peaks and identified 7,021 peaks. Table S2 lists the 7021 peaks in BED format. We used CisGenome [20] to add gene annotations associated with the peaks if a peak is within 50kb from the TSS (transcription start site) of a gene. We were able to annotate 2,487 peaks within 50 kb from TSS of genes. Comparison with a genome-wide p63 ChIP-seq study in human keratinocytes (HFK) [21] showed 46.9% overlap (Table S3) between the two analyses. We also performed functional annotations of the p63 peaks associated genes using DAVID (http://david.abcc.ncifcrf.gov/) [22], [23] for KEGG pathways and Gene Ontology (GO) terms. We found that the GO term cytoskeletal protein binding (GO:0008092) is significantly enriched in the p63 binding targets with FDR <5% (Table 2) with 82 genes related to the cytoskeletal protein binding containing p63 binding sites (Table S4). Other significant enriched biological processes include epidermis development, angiogenesis, ectoderm development and regulation of cell motion. There are 39 p63 targeted genes that are related to regulation of cell motion (Table S5), which could explain p63’s effect on cell migration (Figure 3A) as we observed.

**Table 2:** p63 gene targets related to gene ontology term based on ChIP-seq.

GENE ONTOLOGY TERM	GENE ONTOLOGY ID	GENE COUNTS	PERCENTAGE	P- VALUE	FDR
cytoskeletal protein binding	GO:0008092	82	4.4	2,80E-06	4,50E-03
epidermis development	GO:0008544	40	2.1	2,20E-06	4,10E-03
angiogenesis	GO:0001525	34	1.8	4,20E-06	7,70E-03
ectoderm development	GO:0007398	40	2.1	1,60E-05	2,90E-02
regulation of cell motion	GO:0051270	39	2.1	1,80E-05	3,30E-02

Functional annotation of p63 peaks associated genes (Table S3) using DAVID (http://david.abcc.ncifcrf.gov/) for KEGG pathways and Gene Ontology (GO) terms.

**Figure 4:**
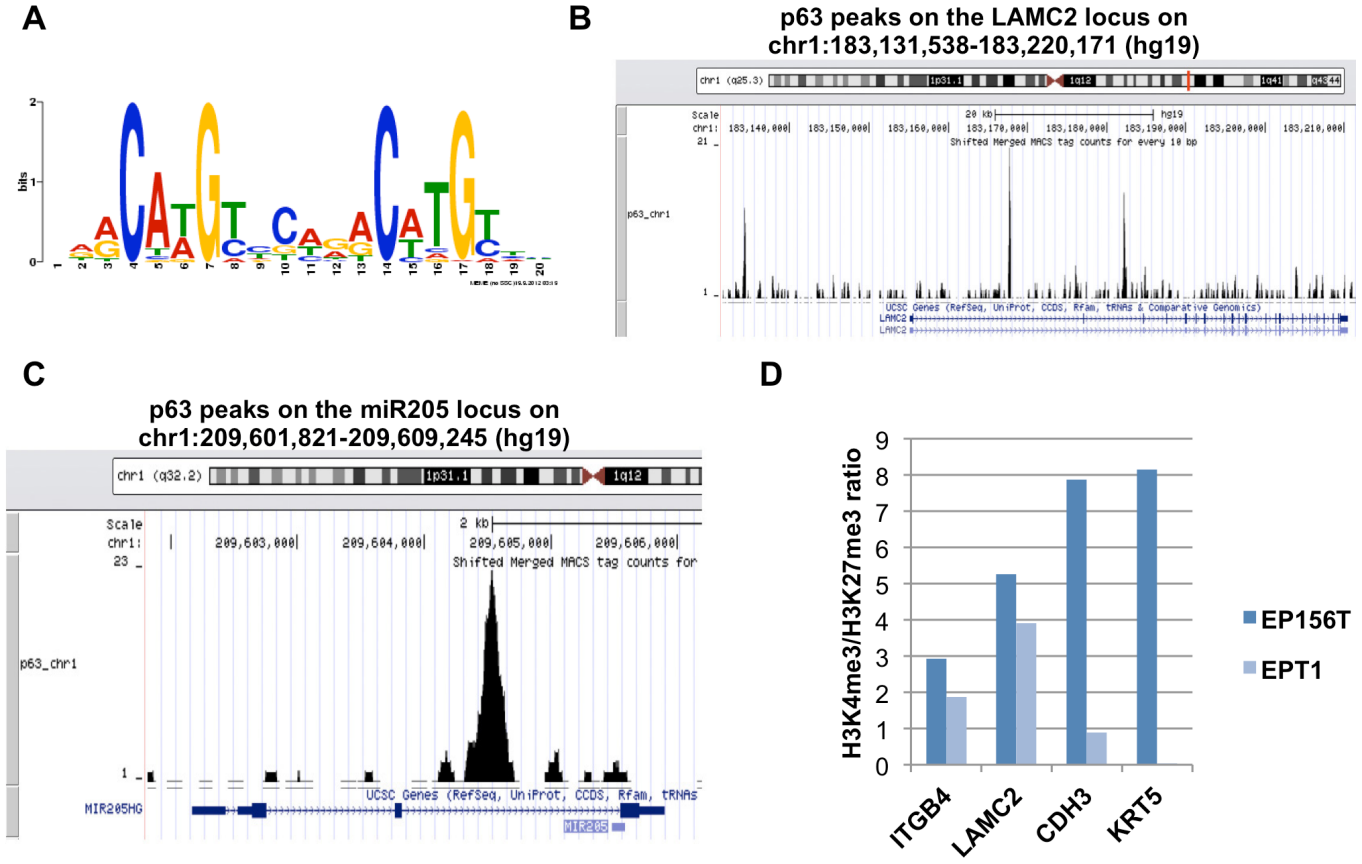
p63 ChIP-seq analysis and epigenetic pattern in EPT1 cells. (A) p63 binding consensus sequence. (B) p63 peak pattern in putative regulatory regions of LAMC2, corresponding to the p63 binding consensus sequence. (C) Peaks corresponding to p63 binding sites within the miR-205 regulatory regions. (D) H3K4me3/H3K27me3 ratio based on ChIP-chip data in EP156T and EPT1 cells.

In order to identify consensus binding motifs for p63 binding in prostate cells, we employed the MEME web server (http://meme.nbcr.net/meme/). We only chose those p63 peaks with FDR equal to zero. After MEME analysis, we identified a consensus motif, which is the same as previously reported for primary human keratinocytes [24]. This motif (Figure 4A) was found to exist in 351 of the 526 selected peaks (FDR  = 0) and has a p-value of 1.5e-717.

Among 4028 genes changed at least 2 fold between EP156T and EPT1 cells in a t-test, 366 genes (9.1%) contained significant p63 peaks in EP156T cells. SAM analysis showed that 70 of the 366 genes (19%) were re-induced at least 2-fold with an FDR <5% in EPT1ΔNp63α cells compared to EPT1 mock transduced cells (EPT1mock), including LAMC2 and ITGA6, and both are p63 targets (Table S3). Additional analyses of genome-wide gene expression data files of both EPT1B8 and EPT2 cells transduced with ΔNp63α and parallel mock-transduced cells showed that ΔNp63α induced re-expression of 57 of the 366 genes (16%) in EPT1B8ΔNp63α cells and 72 of the 366 genes (20%) in EPT2ΔNp63α cells (Table S6, S7, S8). Figure 4B shows the p63 binding peaks found in putative regulatory regions of LAMC2.

We have identified several interesting p63 targeted genes including keratin family genes (KRT5, KRT2, KRT24, KRT8, KRT74, KRT82 and KRT83), laminin family members (LAMC2, LAMB1, LAMB3 and LAMB4), kallikrein family gene KLK4,

ETS family genes (ETS1 and ETV6), fibroblast growth factor family genes (FGF1 and FGF9) and its receptor gene (FGFR2), integrin family genes (ITGA2, ITGA6 and ITGB8) (Table S3, S9).

Finally, Gene Ontology (GO) analyses were performed on genes that were up-regulated after re-expression of ΔNp63α in EPT1B8, EPT1 and EPT2 and that were in the group of the 366 genes defined above. These analyses clearly showed that terms associated with cell adhesion were among the most enriched (Table S6, S7, S8).

### Examination of the potential of E-cadherin to contribute to MET in the present model

In several published EMT models, re-expression of E-cadherin (CDH1) is sufficient to reverse EMT (induce MET) alone or together with co-factors [25]. Interestingly, CDH1 was among the cell adhesion genes that seemed not to be induced by ΔNp63α expression in EPT1B8 cells. As published previously, CDH1 was down-regulated more than 10 fold, but was still easily detectable by Western blot in EPT1 cells following EMT of EP156T cells [8]. In EPT2 cells, derived by their ability to form foci in monolayers and to grow in soft agar in contrast to EPT1 cells, CDH1 production was not detectable [9]. Examination of the clone EPT1B8 showed substantial and variable endogenous expression of CDH1. But this residual endogenous expression of CDH1 was evidently not sufficient in addition to exogenous ΔNp63α expression in order to induce a complete MET. For this reason CDH1-expression was increased to similar levels in EPT1 cells as in EP156T cells by lentiviral transduction of the *CDH1* gene. Although both RT qPCR and Western blot analyses verified that CDH1 expression in EPT1 cells reached comparable levels to those detected in EP156T cells (Figure S4A, S4B), neither CDH1 expression alone nor co-expression with ΔNp63α resulted in more complete MET induction than the one obtained by isolated ΔNp63α re-expression (data not shown). The same panels of cell junction genes were induced in both EPT1 cells and EPT2 cells following re-expression of ΔNp63α as in the EPT1B8 subclone (Table S10, S11).

### The role of ZEB1 knock-down in EMT regulation in prostate cells

ZEB transcription factors are known to repress CDH1 expression following direct binding to E-boxes in the CDH1 promoter [26]. In turn, ZEB1 is targeted by the microRNA 200 family [27] and by miR-205 [28], [29] to constitute reciprocal feedback loops. Examination of our p63 ChIP-seq data files revealed peaks corresponding to p63 binding sites within the miR-205 regulatory regions (Figure 4C), suggesting that p63 might induce miR-205 expression. We analysed our microRNA profiling data files of EP156T, EPT1 and EPT1ΔNp63α cells (Table S12). Pronounced down-regulation of all miR-200 family and miR-205 was observed following EMT. In particular, miR-205 was abundantly expressed in EP156T cells, but undetectable in EPT1 cells. A significant induction of miR-205 was observed in EPT1ΔNp63α cells, although to a much lower level than in EP156T cells.

When EP156T cells underwent EMT to become EPT1 cells, the expression of several classical EMT associated transcription factors, in particular ZEB1, ZEB2, SNAI1 and TWIST2, were strongly increased [8]. In order to examine the MET-inducing potential in our model ZEB1 was knocked down in EPT1B8 and in EPT1B8ΔNp63α cells. Gene expression profiling showed 3.1 fold and 2.6 fold decrease in ZEB1 expression in EPT1B8 and in EPT1B8ΔNp63α cells, respectively, according to RT qPCR validation of microarray results. The knock-down of ZEB1 was associated with an increase of CDH1, ITGB4 and LAMC2 (Figure S4C). ZEB1 knock-down in EPT1B8 cells was additionally associated with specific re-expression of miR-141 and miR-200c, but neither with re-expression of other miR-200 family members nor with re-expression of miR-205 (Table S12), consistent with previous studies in different models [30]. Following ZEB1 knock-down miR-141 and miR-200c were found to be re-expressed in EPT1B8 cells on the average to 14.4% and 13.2%, respectively, of EP156T expression levels (Figure S4D). Still a complete MET based upon cell morphology criteria, was not achieved.

### Epigenetic patterns of ΔNp63α target gene promoters

We have previously published that extensive epigenetic alterations occurred when EP156T cells underwent EMT to become EPT1 cells [31]. As we observed that ΔNp63α re-expression to cell-physiological level was not sufficient to make EPT1B8 cells undergo an EMT and that several of the p63 target genes involved in cell adhesion did not reach expression levels seen in EP156T cells, we speculated that there was an epigenetic restriction reducing the effect of p63 on these promoters. Taking advantage of this dataset, we compared the pattern of transcriptionally stimulatory and inhibitory epigenetic marks on selected p63 target gene promoters. *LAMC2* and *ITGB4* displayed H3K4me3 and H3K27me3 patterns in EPT1 cells similar to that observed in EP156T cells suggesting that the genes are open for transcription also in EPT1 cells. Still, p63 re-expression could not bring the expression of these genes to levels observed in EP156T cells. *KRT5* and *CDH3* have a more repressive H3K4me3/H3K27me3 status in EPT1 cells and interestingly these genes also were clearly up-regulated in EPT1 cells (Figure 4D).

## Discussion

We have examined the role of p63 in EMT and its reversal, MET, since p63 was the most down-regulated transcription factor during EMT in our cell culture model of step-wise accumulation of malignant features in immortalized primary prostate cells. Although loss of p63 was a significant event during EMT, questions remain as to whether it has a functional role in this process or if the expressional changes are secondary to other processes. In order to further elucidate this, we performed an over-expression experiment of the predominant epithelial isoform ΔNp63α in mesenchymal EPT1B8, EPT1 and EPT2 cells and knock-down of p63 in epithelial EP156T cells.

Re-expression of ΔNp63α in EPT1B8, EPT1 and EPT2 mesenchymal like cells and knock-down of p63 in EP156T cells led to widespread and concerted gene expression changes, in particular concerning cell adhesion associated genes. Interestingly, this applies to both cell-to-cell adhesion genes and to genes involved in cell-to-extracellular-matrix (ECM) contact. Earlier studies have shown that p63 plays an important role in regulating cell-adhesion networks in other epithelia [14], [32]. We here show that p63 is an important regulator of such genes in benign immortalized prostate epithelial cells, with ChIP-seq data corroborating our gene expression findings by defining genes directly regulated by p63 promoter binding. Additionally we show that p63 has the ability to regulate these genes in prostate cells with mesenchymal features indicating that the epigenetic context in mesenchymal prostate cells is permissive to p63 binding or that ΔNp63α itself can alter the epigenetic permissibility and result in subsequent p63-dependent gene regulation. We also found evidence of a functional role as EPT1B8 cells’ ability to bind certain ECM components was increased after ΔNp63α re-expression. Additionally, when we knocked down p63 expression in EP156T cells we found that adhesion to the same ECM components decreased. Furthermore, we observed that cell migration was strongly inhibited by forced ΔNp63α expression in EPT1B8 cells, suggesting loss of a mesenchymal trait.

Taking into account that forced expression of ΔNp63α reaches cell-physiological levels and is similar to the p63 expression level seen in EP156T cells, it is striking that the p63 target genes and miR-205 are up-regulated but do not reach the expression levels observed in epithelial EP156T cells, indicating a quantitative rather than qualitative restriction of target gene expression. The limitation of target gene expression is underscored by the observation that neither expression/co-expression of physiological levels of E-cadherin nor complementing knock-down of ZEB1 was sufficient for complete MET induction in EPT cells. The reason for the limited expression of p63-induced cell adhesion and miR-205 genes is unclear. In fact, potential positive feedback circuits can be envisaged according to which p63-mediated induction of miR-205 would lead to ZEB1 reduction (as observed) that next could be expected to alleviate ZEB1 mediated repression of the p63 gene promoter [28]. Among additional reported targets of miR-205, however, is a group of de-ubiquitinating enzymes whose inhibition is associated with increased ubiquitination and proteasomal degradation of p63 [28]. It is conceivable that a balance between these positive feedback and negative feedback mechanisms (or additional undefined ones) could lock ΔNp63α expressing mesenchymal cells at an intermediate stage of MET, such as is observed in our experimental model.

One alternative explanation of quantitative limitation of p63 target gene expression in EPT cells could be related to changes in the epigenetic promoter patterns. Indeed, we have previously shown that EMT results in widespread epigenetic changes between epithelial EP156T cells and mesenchymal EPT1 and EPT2 cells and that these changes are correlated to changes in gene expression, for example for a number of cell adhesion genes [31]. There is limited knowledge concerning which epigenetic backgrounds are favorable to EMT/MET reprogramming, but evidence for this connection has recently emerged also in the field of induced pluripotency [33], [34].

p63 has also been implicated in control of EMT/MET in other studies. Recently, Coppola *et al*. showed that RWPE-1 cells, that are heterogeneous immortalized prostate epithelial cells and display both basal and luminal characteristics, gained mesenchymal features including increased vimentin and decreased E-cadherin expression, when all p63 isoforms where knocked down. In RWPE-2 cells that are more mesenchymal, forced ΔNp63 expression led to decreased vimentin expression [35]. This study suggests that p63 is important to maintain the epithelial phenotype in prostate epithelial cells and that over-expression of ΔNp63 isoforms can reverse mesenchymal features supporting our findings. Even though p63 knock-down led to lower levels of CDH1 (data not shown) we did not observe gain of a mesenchymal phenotype suggesting that EP156T cells are more refractory to induction of mesenchymal features. Another p63 isoform, ΔNp63γ, has been implicated in induction of EMT in human mammary cells [12]. In cells devoid of ΔNp63α and ΔNp63β, ΔNp63γ expression was sufficient to induce EMT in a TGFβ-dependent manner, through ΔNp63γ induced transcription of TGFβ-isoforms. This finding does not seem to apply to our prostate cells as strong re-expression of ΔNp63α did not induce MET, suggesting ΔNp63γ plays a minor role in maintaining the EMT phenotype of the EPT1B8 cells and that p63 isoform function is highly context-dependent.

As we have different passages of EPT1 and EPT1B8 cells with different CDH1 expression levels as well as EPT1 cells with CDH1 over-expression alone and in combination with ΔNp63α, we have a good model to investigate the role of CDH1 in MET. In contrast to other studies [25], CDH1 expression levels did not seem to alter the phenotype further toward an epithelial state, as well as showing that the effect of p63 on cell adhesion genes is CDH1 independent.

To conclude, ΔNp63α re-expression in EPT1B8 cells leads to several functional and expressional changes indicating a switch towards an epithelial phenotype, but lack of re-expression of central epithelial markers and of a clear epithelial morphology suggest that ΔNp63α is only able to induce a partial MET in EPT1B8 cells. Knock-down of p63 in epithelial EP156T cells further supports these findings. Additionally, we show that p63 is functional in a mesenchymal context (i.e. EPT1B8, EPT1 and EPT2 cells) and that this is independent of CDH1 expression. These data suggest that EMT and MET seem to be the extremes of a continuum of states and that certain phenotypes associated with EMT/MET can be controlled without committing fully to this switch.

## Materials and Methods

### Cell Lines

The prostate cell lines EP156T [36], EPT1, EPT1B8 and EPT2 were grown in MCDB153 medium (Biological Ind. Ltd., Israel) with 1% for EP156T and 5% fetal calf serum (FCS) for the others, and supplemented with growth factors and antibiotics as described elsewhere [8], [36]. DNA microsatellite validation of progeny identity of EP156T, EPT1 and EPT2 cells has been published previously [9]. EPT1B8 is a clone obtained from EPT1 cells by limiting dilution to single cells in 96-well plates. The cells were grown in tissue culture plates in a humidified atmosphere containing 5% CO2 at 37°C. The medium was changed every third day.

### Reagents and Antibodies

Primary antibodies p63 (Cat# ab53039), actin (Cat# ab11003), vimentin (Cat# ab8978) and keratin 5 (Cat# ab53121) from Abcam (Cambridge, UK). E-Cadherin (Cat# BD610182) and ITGB4 (Cat# BD611233) from (BD Transduction Labs., BD Biosciences, San Jose, CA, USA)**.** LAMC2 (Cat# MAB19562) from Millipore (Billerica, MA, USA). GJB2 (Cat# 33-5800) from Invitrogen (Camarillo, CA, USA).

### Cloning and Transduction

ΔNp63α-FLAG in a pcDNA3 vector was obtained from L. Ellisen, Harvard. The L335 N792 attSfipolylinkIREStdTom plasmid was digested with *Xho*I and blunt ends were generated using T4 DNA polymerase (Fermentas Cat# EP0061) and dephosphorylated with calf intestine alkaline phosphatase (Fermentas Cat# EF0341). pcDNA3ΔNp63α was excised using *Bam*HI and *Xho*I, and blunted as above. Ligation was performed using HC DNA Ligase (New England Biolabs, Cat# M0202M). Correct insertion was verified with restriction analysis using *Spe*I (New England Biolobs, Cat# R0133S) and sequencing. Pseudotyped retroviral particles were prepared in HEK293 Phoenix cells. Following retroviral transduction Fluorescence Activated Cell Sorting (FACS) was used to select cells expressing exogenous genes.

For p63 knock-down, a pool of three shRNA lentiviral particles (Open Biosystems, clone IDs: V3LHS_397883, V3LHS_397884 and V3LHS_397885) were used, and similarly for ZEB1 knock-down (Open Biosystems, clone IDs: V3LHS_356184, V3LHS_356186 and V3LHS_356187), viral particles with clone ID RHS-4348 were used as negative control. Cells were transduced with a MOI of 1, and subsequently sorted by FACS using the turboGFP reporter in the inserted sequence. The CDH1 gene in a Gateway pDONR vector (Cat# GC-M0954, GeneCopoeia, Rockville, MD, USA) was cloned into pLenti7.3/V5-DEST Gateway Vector Kit (Invitrogen, Carlsbad, CA, USA). The vector was then sequenced to confirm correct insertion. EPT1 and EPT1ΔNp63α cells were transduced according to the manufacturer’s protocol and cells expressing CDH1 were selected using FACS based on EmGFP expression.

### Real-time quantitative PCR (qPCR)

Total RNA was extracted using the miRNeasy kit from Qiagen (Cat# 217004). The total RNA was DNase treated, ss-cDNA was synthesized and the RT qPCR was run and analyzed as previously described [37], using pre-designed Taqman probes (Table S13).

### Global Gene Expression Analysis

The Agilent Human Whole Genome (4×44 k) Oligo Microarray with Sure Print Technology (Agilent Technologies, Design G4112-60520 G4845-60510, Palo Alto, CA, USA), was used to analyze samples in the present study. Total RNA purification, cDNA labeling, hybridization and normalization have been described previously [37], [38]. Following normalization, significance analysis of microarray (SAM) of the J-Express program package (http://www.molmine.com) [39] was used for identification of differentially expressed genes. Only genes that changed at least 2.0 fold with FDR below 5% were considered as differentially expressed genes in cell lines. For GO analysis, cell adhesion-related GO terms were searched for and a Fischer’s exact test was performed to obtain nominal p-values.

### Micro-RNA profiling

Total RNA was extracted using the miRNeasy kit from Qiagen (Cat# 217004). The miRNA expression was profiled by Toray (Tokyo, Japan) platforms. According to the Toray protocol, 500 ng total RNA was labeled using the miRCURY LNA microRNA Hy5 Power labeling kit (Cat# 208030-A, Exiqon) and hybridized to the 3D-Gene™ Human miRNA oligo chip. The microarray was spin-dried followed by image scanning using the 3D-Gene™ scanner at 635 nm excitation. The obtained images were numerated by 3D-Gene™ Extraction. The detected spots were defined as the ones that had the signal intensity over 2SD plus mean of background signal intensity. The mean of the background-subtracted signal intensity of duplicate spots was used for the further analysis.

### Chromatin immunoprecipitation and ChIP-chip

ChIP was performed according to the Agilent ChIP-chip protocol with modifications as previously described [31]. To immunoprecipitate chromatin, 6×10^7^cells were treated with 1% formaldehyde at room temperature for 10 min followed by quenching with 0.125 M glycine. Cells were lysed and the nuclei were sonicated under conditions yielding DNA fragments ranging from 200 to 800 base pairs. Five percent of the sonicated material was saved as whole cell extract. Sonicated lysate was divided into three equal volumes and immunoprecipitated with specific or non-specific antibody bound to protein A magnetic beads (Invitrogen) overnight at 4°C with rocking. Antibodies used were against: H3K4me3 (Abcam, Cat# ab8580) or H3K27me3 (Abcam, Cat# ab6002) or mouse IgG (Sigma). Five µg of antibody was used per 2×10^7^cells. Immunoprecipitated complexes were collected, washed and eluted using the Dynal Magnetic Particle Concentrator (Invitrogen). Eluted DNA and whole-cell extracts were incubated at 65°C in a rotating incubator for 8 hours to reverse cross-links. DNA samples were sequentially treated with RNase A and proteinase K and then purified by phenol/chloroform extraction. The immunoprecipitated (ChIP’ed) and purified DNA was ethanol precipitated using glycogen as a carrier and resuspended in nucleic acid grade water. Analysis of promoter H3K4me3/H3K27me3 ratio was performed as previously described [31].

### ChIP sequencing (ChIP-seq)

The Chromatin immunoprecipitation was essentially as described for ChIP-chip above. For immunoprecipitation 5 µg of ChIP-grade p63 antibody (Abcam, Cat# ab3239) was used per 2×10^7^cells.

### Chip-seq Data analysis

After chromatin immunoprecipitation and sequencing, raw reads were mapped to the human genome (hg19) with bowtie [18] using the default parameters (-S -l 28 -n 2 -e 70 -m 1). MACS program [19] was used to find peaks with the parameters (p-value: 1.00e-05, FDR: 10%, model fold: 10-30, effective genome size: 2.70e+09). The genome annotation was performed with the CisGenome program [20]. Motif scanning was performed with the MEME web server (http://meme.nbcr.net/meme/). To scan for p63 binding sites in the regulatory regions of genes, we use the Python script (downloaded from http://131.174.221.43/bioinfo/p53scan/) developed by Kouwenhoven *et al*. in their genome wide analysis of p63 binding sites [24] with the PMW profile for p63.

### Indirect immunofluorescence (IF) and Western blot assays

For IF cells were grown on 10 mm Assistant glass coverslips in 24 well plates, then washed with PBS, fixed (4% fresh formaldehyde in PBS for 20 min. at room temperature), permeabilized (0.5% Triton X-100 for 10 min.), blocked (100 mM glycin for 10 min) and stored (in PBS at 4°C) with PBS wash between each step. Following blocking with 0.5% BSA/PBS for 15 min. primary mouse monoclonal antibodies were added at room temperature for 1hour at indicated dilutions in 0.5% BSA/PBS. The FITC-labelled secondary anti-mouse Ig (SouthernBiotech, Birmingham, AL, USA, Cat# 4050-02) was added for 1 h at room temperature in 0.5% BSA/PBS. Coverslips were mounted in Prolong Gold with DAPI (Molecular Probes, Invitrogen, Carlsbad, CA, USA) on glass slides and analysed using Leica TCS SP5 confocal microscopy or Leica DM IRBE fluorescence microscopy.

For WB analysis cells were lysed in Lysis Buffer 6 (R&D Systems Inc, Minneapolis, MN, USA, Part# 895561) and Protease Inhibitor Cocktail Set I diluted 1∶100 (Calbiochem, Cat# 535142). Protein concentrations were measured using the Bio-Rad DC protein assay (Biorad Labs, Hercules, CA, USA, Cat# 500-0112), and 3,75 µg protein lysates were separated by 10% polyacrylamide gel (Biorad Labs, Hercules, CA, USA, Cat# 161-1173) SDS electrophoresis followed by blotting to PVDF membranes (GE Healthcare Life Sciences, Uppsala, Sweden, Cat# RPN1416F). Membranes were blocked for one hour in PBS 0,1% Tween and 5% blotting grade non-fat dry milk (Bio-Rad, Cat#170-6404). Primary antibodies were incubated for 1 hour in blocking buffer at RT were, and HRP-labelled secondary antibodies (GE Healthcare, Cat# NA931V, NA934V), were incubated as the primary antibodies 1/8000. ECL plus western blotting detection reagents (GE Healthcare, Cat# RPN2132) was used for detection with Chemidoc XRS using Quantity One 4.6.5 (Bio-Rad). Molecular weight marker used was ECL DualVue Marker (GE Healthcare, Cat# RPN810).

### Migration and invasion assay

For trans-well migration assays and invasion assays, 1.8×10^5^ cells starved overnight in serum-free medium were added to the top chambers of 24-well trans-well plates (8 mm size, Cat# CBA-100-C, Cell Biolabs Inc.) and media containing 10% fetal calf serum (FCS) were added to the bottom chambers. Incubation was for 12 h in the migration assay and 24 h in the invasion assay. Top (non-migrating) cells were removed, bottom (migrating) cells were stained and absorbance was recorded at 560 nm. All of these assays were done in at least triplicate and the data are presented as the average absorbance of migrating cells and invading cells ±s.d.

### Cell adhesion, anoikis and apoptosis assay

1.0×10^5^ cells were seeded in each well of a 48 well plate, incubated at 37°C and 5% CO_2_ for 90 minutes. The cells were then washed and treated according to the manufacturer’s protocol. CytoSelect™ 48-Well Cell Adhesion Assay (ECM Array, Colorimetric Format, Cat# CBA-070, Cell Biolabs Inc., San Diego, CA, USA). Anoikis was assayed by seeding 20.000 EPT1B8 cells and 10.000 EP156T cells per well in a 96 well plate covered with hydrogel and a in normal control plate. The cells were incubated for 48 h before the wells were assayed by MTT solution for viability according to the manufacturer’s protocol. CytoSelectTM 96-Well Anoikis Assay (Cat# CBA-081, Cell Biolabs Inc., San Diego, CA, USA), Apoptosis was determined using the Caspase-Glo 3/7 Assay (G8090, Promega, Madison, WI, USA) according to the manufacturer's protocol. Briefly, 10.000 EPT1B8 cells and 5000 EP156T cells were plated in 96-well plates and incubated for 12 h followed by staurosporine (Sigma, S6942) treatment at indicated concentrations for 6 h at 37°C to induce apoptosis. Equal volumes of Caspase-Glo 3/7 Reagent was added directly to each well and the plates were incubated at room temperature for 1 h before recording luminescence in a Synergy H1 reader (BioTek Instruments Inc., Bad Friedrichshall, Germany).

### Anchorage-independent growth assay

The anchorage independent growth was examined in soft agar. 50 ml of base agar matrix (CytoSelect™ Cell, Colorimetric kit, Cat# CBA-135, Cell Biolabs Inc., San Diego, CA, USA) was added in the bottom of each well of a 96-well plate. When the agar was solid, 75 ml of cell suspension/soft agar matrix containing 2500 cells was layered on top followed by 50 ml of complete medium. After 8 days of incubation, the agar matrix was solubilized and cells stained and absorbance was recorded at 570 nm. Data show the quantification of proliferation of EPT1B8 cells and positive control EPT2 cells in the soft agar assay.

### Accession numbers of gene expression, ChIP-chip and ChIP-seq data

Performed as described previously [31]. Annotated microarray data were uploaded in the BASE database, formatted and exported to ArrayExpress (http://www.ebi.ac.uk/arrayexpress) at the European Bioinformatics Institute in agreement with the MIAME guidelines, accession number: E-MTAB-1471. The ChIP-chip study has ArrayExpress accession numbers: E-TABM-635; E-TABM-983 [31]. The ChIP-seq data discussed in this publication have been deposited in NCBI's Gene Expression Omnibus [40] and are accessible through GEO Series accession number GSE43111 (http://www.ncbi.nlm.nih.gov/geo/query/acc.cgi?acc=GSE43111).

## Supporting Information

Figure S1 (A) Schematic overview of p63 isoforms adopted from Mangiulli et.al. [2]. There are two different 5’ variants ΔN and TA which can combine with five 3’ variants α,β,γ,δ and ε, giving 10 isoforms all together. (B) Schematic overview of the construct used to over-express ΔNp63α in EPT cells. (C) Schematic figure of the cell culture model. EP156T cells are immortalized cells derived from primary benign prostate epithelial cells. EPT1 cells are EP156T cells that have undergone an EMT [8]. EPT2 cells have acquired ability to grow anchorage independently and are derived from EPT1 cells [9].
(TIFF)

Figure S2 (A) Invasion of EPT1B8 ΔNp63α and EPT1B8 as measured by invasion through a Boyden chamber inserted with extracellular matrix. Student’s t-test was used for statistical analysis (p = 0.098). (B) Induction of apoptosis in EPT1B8 ΔNp63α and EPT1B8 by staurosporine measured by caspase 3/7 activity. Error bars show ±s.d (C) Immunofluorescence by phalloidin staining actin filaments in EPT1B8 ΔNp63α and EPT1B8 cells.
(TIFF)

Figure S3 (A) qRT-PCR and (B) Western Blot of EP156T p63 knock-down (p63KD) and EP156T showing relative p63 expression. Error bars show ±s.d. (*, p<0.01) (C) qRT-PCR of genes involved in cell adhesion ITGB4, LAMC2, CDH3 and KRT5 in EP156T p63 knock-down (p63KD) and EP156T. (D) Boyden chamber migration assay of EP156T p63KD and EP156T cells. (n.s, p = 0.49). (E) Invasion of EP156T p63KD and EP156T as measured by invasion through a Boyden chamber inserted with extracellular matrix. (**, p = 0.001). (F) Induction of apoptosis in EP156T p63KD and EP156T by staurosporine measured by caspase 3/7 activity. (G) EP156T p63KD and EP156T cells grown on a hydrogel covered wells (anoikis) and regular wells, cells alive stained after 24 hours. (*, p<0.01). Error bars show ±s.d. of at least three replicates. Student’s t-test was used for statistical analyses.
(TIFF)

Figure S4 (A) qRT-PCR and (B) Western Blot of EPT1 cells with CDH1 overexpression (EPT1 CDH1) compared to control (EPT1) and EP156T showing comparable CDH1 expression in EPT1 CDH1 and EP156T. Error bars show ±s.d. (C) Knock-down of ZEB1 in EPT1B8 and associated increase of CDH1, ITGB4 and LAMC2 assayed by qRT-PCR. Error bars show ±s.d. (D) miR-141 and miR-200c expression in EPT1B8 cells following ZEB1 knock-down compared to levels in EP156T cells. (*n.d; not detected in EPT1B8 cells).
(TIFF)

Table S1 **Relative enrichment of GO-terms related to cell adhesion in EP156T p63 knock-down (p63KD) compared to EP156T, using specific search for terms for different cell adhesion complexes.** P-values are nominal and calculated by Fischer’s exact test.
(XLSX)

Table S2 **List of 7021 binding peaks called with MACS after p63 ChIP-seq in EP156T cells in BED format.**
(BED)

Table S3 **Annotated p63 peaks from ChIP-seq using CisGenome within 50 kb from the TSS (transcription start site) of a gene.** These data are integrated with ChIP-seq data from Human Foreskin Keratinocyte (HFK) cells after McDade et al. [21].
(XLS)

Table S4 **Genes belonging to the GO term cytoskeletal protein binding (GO:0008092) found to be significantly enriched in p63 binding targets with 82 genes related to the cytoskeletal protein binding containing p63 binding sites.**
(XLSX)

Table S5 **Genes associated with p63 binding sites that are related to regulation of cell motion (GO:0051270).**
(XLSX)

Table S6 **366 genes that were differentially expressed (>2 fold between EP156T and EPT1) and had significant p63 peaks in EP156T found by ChIP-seq analysis, were compared to differentially expressed genes (>2-fold) between EPT1ΔNp63α and EPT1mock cells (Table S6).** Genes that were in both groups were analysed by functional annotation by DAVID (http://david.abcc.ncifcrf.gov/).
(XLS)

Table S7 **366 genes that were differentially expressed (>2 fold between EP156T and EPT1) and had significant p63 peaks in EP156T found by ChIP-seq analysis, were compared to differentially expressed genes (>2-fold) between EPT1B8ΔNp63α and EPT1B8mock.** Genes that were in both groups were analysed by functional annotation by DAVID (http://david.abcc.ncifcrf.gov/).
(XLS)

Table S8 **366 genes that were differentially expressed (>2 fold between EP156T and EPT1) and had significant p63 peaks in EP156T found by ChIP-seq analysis, were compared to differentially expressed genes (>2-fold) between EPT2ΔNp63α and EPT2mock.** Genes that were in both groups were analysed by functional annotation by DAVID (http://david.abcc.ncifcrf.gov/).
(XLS)

Table S9 **Examples of relevant genes with the p63 consensus binding sites in the regulatory regions.**
(XLSX)

Table S10 **Relative enrichment of GO-terms related cell adhesion in EPT1 ΔNp63α compared to EPT1, using specific search for terms for different cell adhesion complexes.** P-values are nominal and calculated by Fischer’s exact test.
(XLSX)

Table S11 **Relative enrichment of GO-terms related cell adhesion in EPT2 ΔNp63α compared to EPT2, using specific search for terms for different cell adhesion complexes.** P-values are nominal and calculated by Fischer’s exact test.
(XLSX)

Table S12 **Normalized expression values of microRNA in; (A) EP156T, EPT1 and EPT1 ΔNp63α. (B) EPT1B8, EPT1B8 ΔNp63α, EPT1B8 ZEB1 knockdown and EP156T.**
(XLSX)

Table S13 **RT-qPCR assays and catalog numbers (Applied Biosystems).**
(XLSX)
