# Supplementary figures and images for: p63 Attenuates Epithelial to Mesenchymal Potential in an Experimental Prostate Cell Model
Source: PLoS One. 2013 May 1;8(5):e62547. doi: 10.1371/journal.pone.0062547 (PMC3641034; doi:10.1371/journal.pone.0062547)

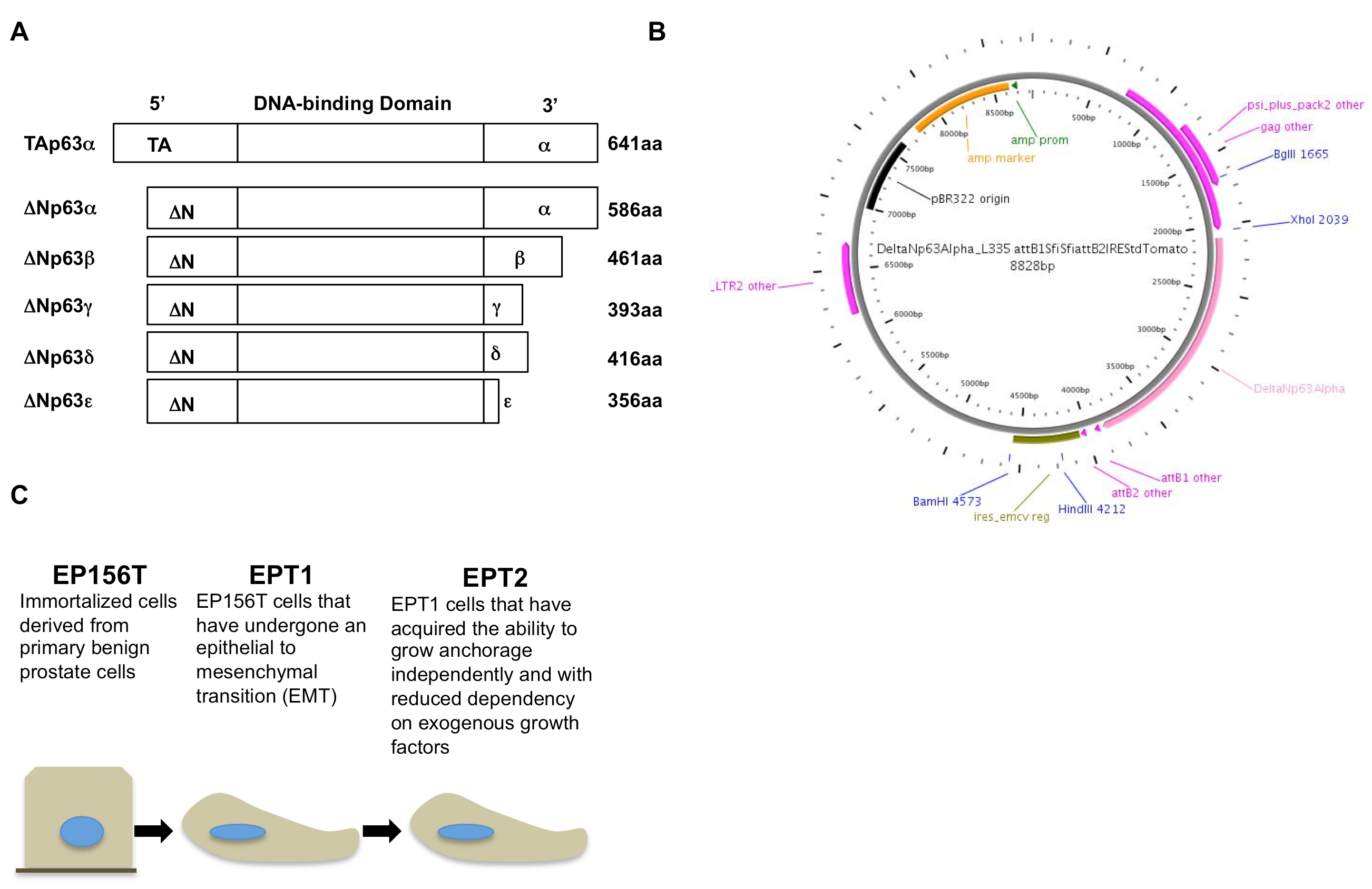

Supplement: Figure S1 — (A) Schematic overview of p63 isoforms adopted from Mangiulli et.al. [2]. There are two different 5’ variants ΔN and TA which can combine with five 3’ variants α,β,γ,δ and ε, giving 10 isoforms all together. (B) Schematic overview of the construct used to over-express ΔNp63α in EPT cells. (C) Schematic figure of the cell culture model. EP156T cells are immortalized cells derived from primary benign prostate epithelial cells. EPT1 cells are EP156T cells that have undergone an EMT [8]. EPT2 cells have acquired ability to grow anchorage independently and are derived from EPT1 cells [9]. (TIFF) [file pone.0062547.s001.tif]

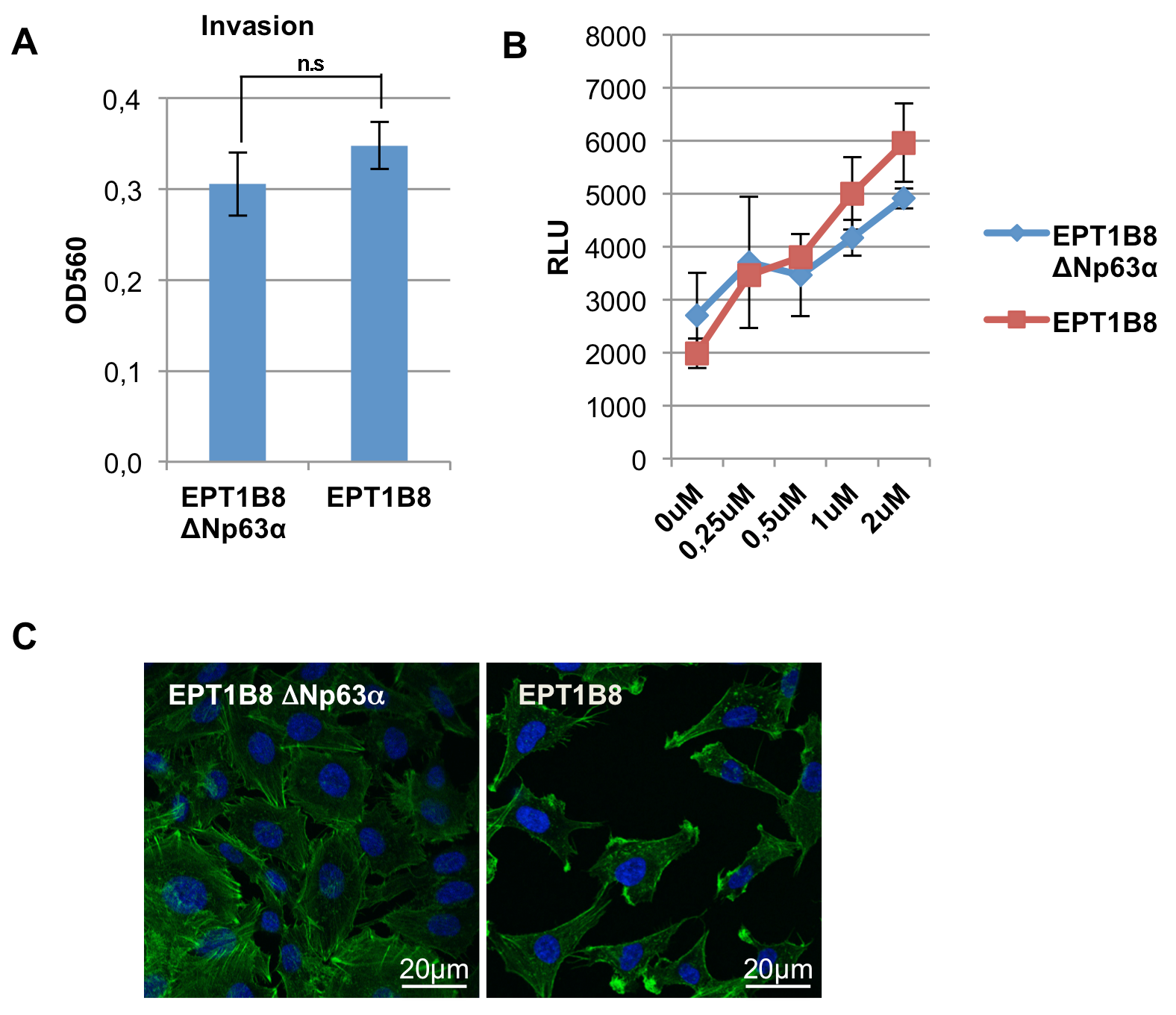

Supplement: Figure S2 — (A) Invasion of EPT1B8 ΔNp63α and EPT1B8 as measured by invasion through a Boyden chamber inserted with extracellular matrix. Student’s t-test was used for statistical analysis (p = 0.098). (B) Induction of apoptosis in EPT1B8 ΔNp63α and EPT1B8 by staurosporine measured by caspase 3/7 activity. Error bars show ±s.d (C) Immunofluorescence by phalloidin staining actin filaments in EPT1B8 ΔNp63α and EPT1B8 cells. (TIFF) [file pone.0062547.s002.tif]

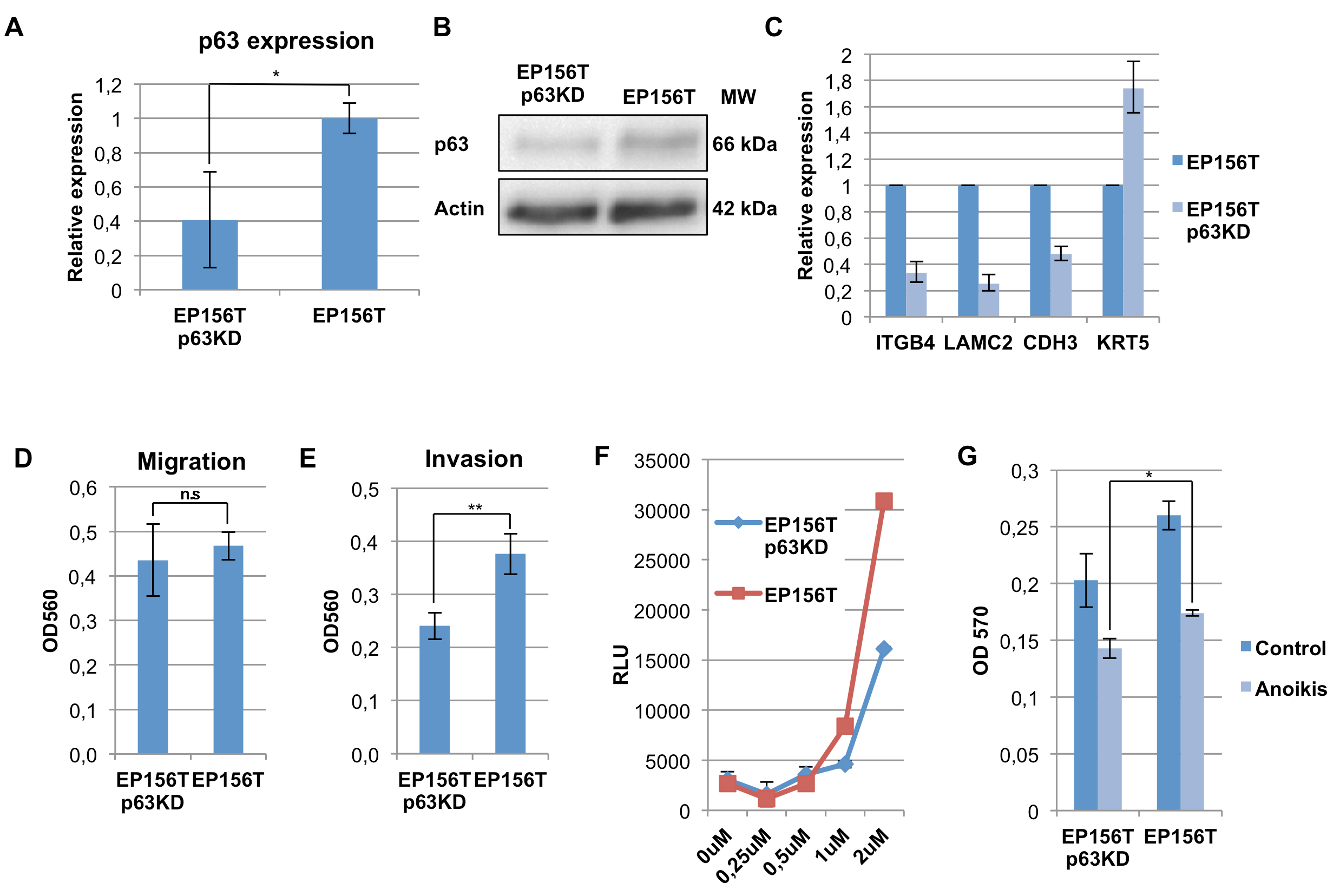

Supplement: Figure S3 — (A) qRT-PCR and (B) Western Blot of EP156T p63 knock-down (p63KD) and EP156T showing relative p63 expression. Error bars show ±s.d. (*, p<0.01) (C) qRT-PCR of genes involved in cell adhesion ITGB4, LAMC2, CDH3 and KRT5 in EP156T p63 knock-down (p63KD) and EP156T. (D) Boyden chamber migration assay of EP156T p63KD and EP156T cells. (n.s, p = 0.49). (E) Invasion of EP156T p63KD and EP156T as measured by invasion through a Boyden chamber inserted with extracellular matrix. (**, p = 0.001). (F) Induction of apoptosis in EP156T p63KD and EP156T by staurosporine measured by caspase 3/7 activity. (G) EP156T p63KD and EP156T cells grown on a hydrogel covered wells (anoikis) and regular wells, cells alive stained after 24 hours. (*, p<0.01). Error bars show ±s.d. of at least three replicates. Student’s t-test was used for statistical analyses. (TIFF) [file pone.0062547.s003.tif]

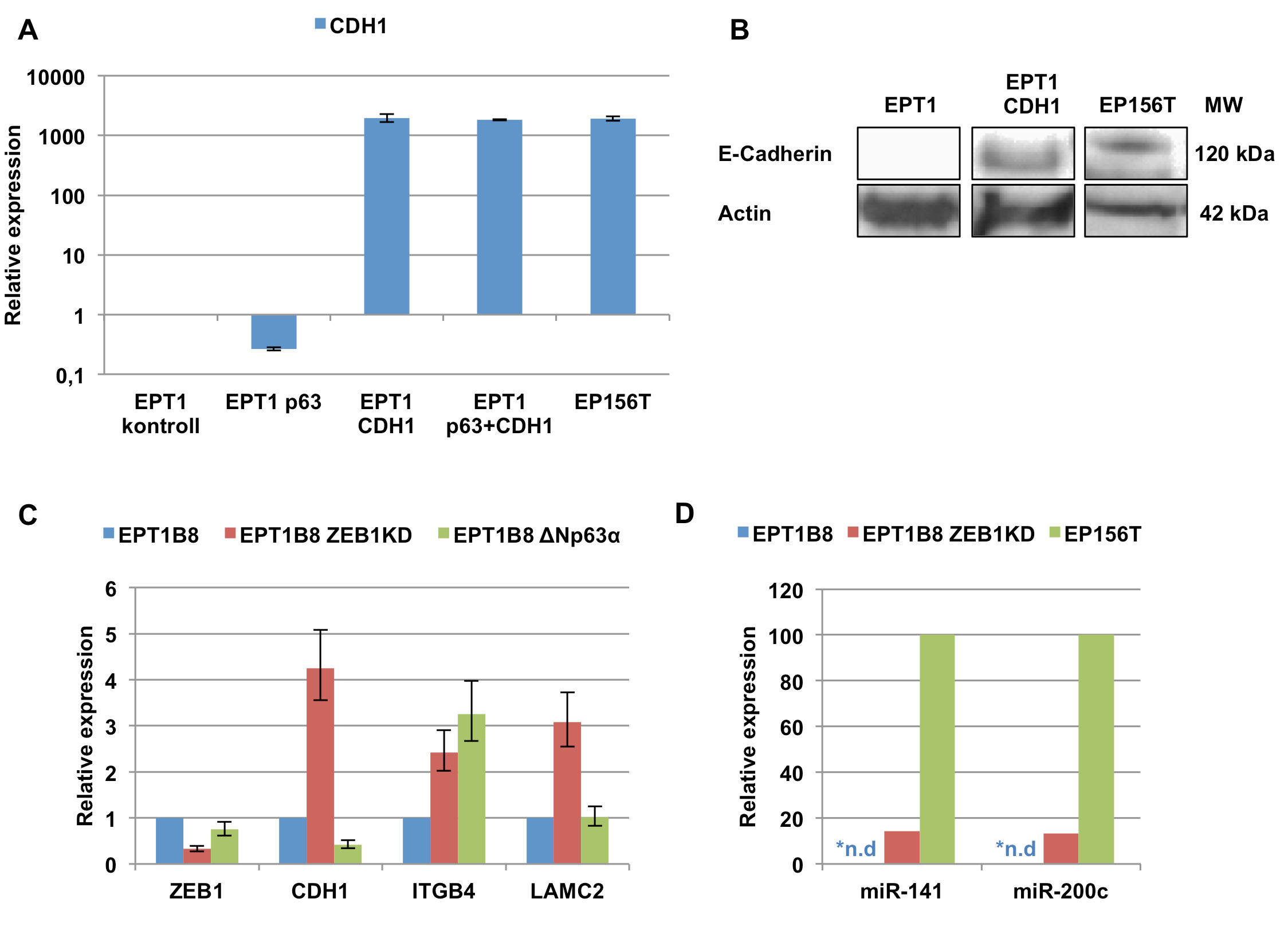

Supplement: Figure S4 — (A) qRT-PCR and (B) Western Blot of EPT1 cells with CDH1 overexpression (EPT1 CDH1) compared to control (EPT1) and EP156T showing comparable CDH1 expression in EPT1 CDH1 and EP156T. Error bars show ±s.d. (C) Knock-down of ZEB1 in EPT1B8 and associated increase of CDH1, ITGB4 and LAMC2 assayed by qRT-PCR. Error bars show ±s.d. (D) miR-141 and miR-200c expression in EPT1B8 cells following ZEB1 knock-down compared to levels in EP156T cells. (*n.d; not detected in EPT1B8 cells). (TIFF) [file pone.0062547.s004.tif]
